# EHMTI-0247. Headache-attributed lost productivity, and the influence of headache frequency, in two different female workforces in Turkey

**DOI:** 10.1186/1129-2377-15-S1-B31

**Published:** 2014-09-18

**Authors:** M Selekler, S Dundar-Yilmaz, C Ozerdem, TJ Steiner

**Affiliations:** 1 Department of Neurology, Kocaeli University Faculty of Medicine, Kocaeli, Turkey; 2 Department of Neuroscience, Norwegian University of Science and Technology, Trondheim, Norway

## Background

In our previous workforce study at Ford Otomotiv Sanayi (FO), the association between high headache frequency and presenteeism in the relatively small female workforce attracted our attention.

## Aim

We compared this workforce with another female hospital-based workforce to explore similarities and dissimilarities.

## Methods

The HALT-30 questionnaire had been employed as the survey instrument at FO. We administered the same to nurses and female residents of Kocaeli University Medical Faculty Hospital (KUMFH). We categorised headache frequency into four groups: low (≤1/month), moderate (2-4/month), high (5-14/month) and headache on ≥15 days/month.

## Results

At FO (n=431; mean age 29.2±4.5 years), 1-month headache prevalence was 62.6%, and at KUMFH (n=466; mean age 29.9±4.9 years) it was 76.8% (p<0.05). Distributions between the frequency groups were similar: 16.6%, 46.3%, 31.5%, 5.5% (low to high) at FO and 16.4%, 52.7%, 25.1%, 6.8% at KUMFH (although p<0.05). Of those with headache, 135 (50%) at FO and 145 (42.9%) at KUMFH reported lost productivity, mostly from presenteeism. The key finding was a clear gradient associating headache frequency and presenteeism at individual level in both workforces (FO: 1.0, 2.0, 3.9, 10.3; KUMFH: 1.0, 1.9; 3.5, 6.0 days/month), but not absenteeism (FO: 0.2, 0.02, 0.05, 0; KUMFH: 0, 0.02; 0, 0.07 days/month).

## Discussion

Headache frequency and lost productivity expressed as presenteeism are high among working women. The association between them is expected. Turkish employment situation might be a factor in determining absenteeism rates. People with infrequent attacks, facing one unexpectedly, might be unprepared for avoiding absenteeism.

No conflict of interest.

